# Skeletal muscle satellite cell-derived mesenchymal stem cells ameliorate acute alcohol-induced liver injury

**DOI:** 10.7150/ijms.68971

**Published:** 2022-01-24

**Authors:** Jae Sik Chung, Soonjae Hwang, Ju Eun Hong, Minjeong Jo, Ki-Jong Rhee, Seongyup Kim, Pil Young Jung, Youngdae Yoon, Seong Hee Kang, Hoon Ryu, Moon Young Kim, Keum Seok Bae, Young Woo Eom

**Affiliations:** 1 Department of Surgery, Yonsei University Wonju College of Medicine, Wonju, Gangwon-do 26426, Republic of Korea.; 2 Regeneration Medicine Research Center, Yonsei University Wonju College of Medicine, Wonju, Gangwon-do 26426, Republic of Korea.; 3 Department of Biochemistry, Lee Gil Ya Cancer and Diabetes Institute, GAIHST, Gachon University College of Medicine, Incheon 21999, Republic of Korea.; 4 Department of Biomedical Laboratory Science, College of Health Sciences, Yonsei University MIRAE Campus, Wonju, Gangwon-do 26493, Republic of Korea.; 5 Mitohormesis Research Center, Yonsei University Wonju College of Medicine, Wonju, Gangwon-do 26426, Republic of Korea.; 6 Department of Internal Medicine, Yonsei University Wonju College of Medicine, Wonju, Gangwon-do 26426, Republic of Korea.; 7 Cell Therapy and Tissue Engineering Center, Yonsei University Wonju College of Medicine, Wonju, Gangwon-do 26426, Republic of Korea.

**Keywords:** skeletal muscle satellite cells, mesenchymal stem cells, binge ethanol, inflammation, liver, gut

## Abstract

Cultured human skeletal-muscle satellite cells have properties of mesenchymal stem cells (skeletal muscle satellite cell-derived mesenchymal stem cells, SkMSCs) and play anti-inflammatory roles by secreting prostaglandin E2 and hepatocyte growth factor (HGF). To evaluate the utility of SkMSCs in treating liver diseases, we determined whether SkMSCs could ameliorate acute liver and gut inflammation induced by binge ethanol administration. Binge drinking of ethanol led to weight loss in the body and spleen, liver inflammation and steatosis, and increased serum ALT and AST levels (markers of liver injury), along with increased IL-1β, TNF-α, and iNOS expression levels in mice. However, levels of these binge-drinking-induced indicators were reduced by a single intraperitoneal treatment of SkMSCs. Furthermore, levels of bacteria-derived lipopolysaccharide decreased in the livers and sera of ethanol-exposed mice after SkMSC administration. SkMSCs decreased the extent of tissue inflammation and reduced villus and crypt lengths in the small intestine after alcohol binge drinking. SkMSCs also reduced the leakage of blood albumin, an indicator of leaky gut, in the stool of ethanol-exposed mice. Alcohol-induced damage to human colonic Caco-2/tc7 cells was also alleviated by HGF. Therefore, a single treatment with SkMSCs can attenuate alcoholic liver damage by reducing inflammatory responses in the liver and gut, suggesting that SkMSCs could be used in cell therapy to treat alcoholic liver diseases.

## Introduction

Alcoholic liver disease can progress from simple hepatic steatosis and steatohepatitis to end-stage organ failure (cirrhosis). Cirrhosis is the tenth leading cause of mortality in developed countries 1. Alcohol consumption is a major cause of this disease - approximately 50% of all cirrhosis-associated mortalities are associated with alcohol consumption 2, 3. Chronic alcohol abusers and patients with alcoholic liver disease exhibit increased serum endotoxin levels and a disrupted gut barrier with increased permeability 4-6. Alcohol-associated liver inflammation is mediated by commensal gut microbe-derived endotoxins, which induce TLR4-mediated inflammatory signaling as well as hepatic steatosis in mice 7-9. Such findings from animal models and alcoholic patients indicate that alcohol-induced liver injury is associated with inflammation of the gut barrier (the intestinal epithelium) that maintains the immunologic homeostasis between the gut tissue and resident microbiota. Alcohol-associated hepatic steatosis and steatohepatitis result from enteric bacteria as germ-free mice display reduced extent of liver inflammation and alcohol-fed mice administered with antibiotics display decreased levels of hepatic steatosis 10-12.

Curative treatment for alcohol-promoted cirrhosis is currently limited to orthotropic liver transplantation. However, a global shortage of donor organs has resulted in the death of patients on the organ waitlist 13. Therefore, stem cell-based therapy has emerged as a promising alternative with accumulating evidence of its efficacy in experimental and clinical studies 14-17. Adult stem cells have been isolated from the bone marrow and identified as mesenchymal stem cells (MSCs) 18. The self-renewal and differentiation abilities of these cells are of great interest in cell-based therapies 17. Alternate sources of MSCs, such as umbilical cord tissue, blood, liver, dental pulp, and skin, have been investigated; the functions of MSCs depend on the collection procedure, cell quantity, immaturity, and cell profile 19, 20. MSCs originating from different sources are known to play a role in healing and repairing damaged tissues through secreted cytokines 21.

Although skeletal-muscle satellite cells (SCs) are important effector cells for the regeneration of damaged muscle, other resident cell types, including motor neurons, endothelial cells, immune cells, and fibro-adipogenic progenitors (FAPs), play a positive role in muscle repair and homeostasis 22-24. The acute inflammatory response after muscle injury induces the infiltration of neutrophils and M1 macrophages 5,6, leading to SC activation 7 and extracellular matrix production by FAPs. FAPs induce the regeneration of damaged muscle tissue and maintain homeostasis in muscle tissue 22, 25. According to a previous study by our group, cultured SCs showed MSC-like phenotypes (skeletal muscle satellite cell-derived mesenchymal stem cells, SkMSCs), including anti-inflammatory effects on macrophages through PGE2 and HGF, leading to the inhibition of IL-1β secretion 26. In addition, the population doubling time of SkMSCs is markedly shorter than that of MSCs isolated from bone marrow or adipose tissue, which is expected to be a great advantage for cell therapy using adult stem cells 26. Endotoxins derived from intestinal microorganisms have been reported to migrate to liver tissue owing to excessive alcohol intake and induce damage in small intestinal epithelial cells, promoting alcohol-associated hepatic steatosis and steatohepatitis through the activation of immune cells, including neutrophils and macrophages, which exacerbates liver injury 27, 28. However, whether adult stem cells can reduce alcohol-induced hepatic and intestinal damage in a mouse model of alcohol-induced liver damage has not been investigated. In this study, we aimed to determine whether SkMSCs could treat alcohol-induced inflammatory liver injury and gut inflammation in mice.

## Materials and Methods

### SkMSC cell culture

This study was approved by the Institutional Review Board of Yonsei University Wonju College of Medicine (CR320308). Human skeletal muscle satellite cells (SCs) at passage (P) 1 were purchased from ScienCell (Cat no. 3510, Carlsbad, CA, USA), seeded in 100-mm dishes coated with poly-L-lysine (ScienCell), and subcultured in skeletal muscle cell medium (SkMCM, ScienCell) supplemented with 2% fetal bovine serum, antibiotics, and skeletal muscle cell growth supplement (SkMCGS, ScienCell). Upon confirmation at P5, the isolated cells showed characteristics of MSCs rather than those of satellite cells 26. Accordingly, P5 cells were used for this experiment.

### Animal treatments

All animal housing and experimental procedures were reviewed and approved by the Institutional Animal Care and Use Committee of Yonsei University at Wonju (YWCI-201909-013-01 and YWC-200305-1). All mice were maintained under controlled lighting (12-h light/dark cycle) with food and water provided *ad libitum*. Age-matched 8-10-week-old female C57BL/6 wild-type (WT) mice were administered a single intraperitoneal (i.p.) injection of 2.0 × 10^6^ SkMSCs/200 μL in Hanks' balanced salt solution (HBSS, Sigma, San Diego, CA, USA) on day 2 after receiving a second binge alcohol dose (6 g/kg/dose). Control mice were administered a vehicle (phosphate-buffered saline [PBS], Gibco BRL, Rockville, MD, USA). Mice were administered three oral doses of binge alcohol (6 g/kg/dose) or dextrose (as control) at 12-h intervals and euthanized at 1 h after the last ethanol treatment.

### Histology

The liver and small intestine (ileum) were excised and fixed in 10% formalin. For microscopic examination, tissues were embedded in paraffin and sectioned (4 μm) using a rotary microtome. Slides were stained with hematoxylin, bluing buffer, and eosin for 1 min each; dehydrated using alcohol (95%, 100%, 100%, and 100%) for 1 min each; and rinsed twice with xylene for 1 min each. Slides were photographed using an optical microscope (Leica, Wetzlar, Germany) and rendered using Adobe Photoshop and Leica software. Histological scoring of the liver was performed based on lobular inflammation and steatosis. Lobular inflammation scores of the liver during binge-induced liver damage were graded blindly by a board-certified pathologist as follows: 0, none; 1, 1-2 foci/×20 fields; 2, 2-4 foci/×20 fields; and 3, >4 foci/×20 fields. Hepatic steatosis, characterized by hepatocytes containing fat vacuoles, was subjectively visualized by hematoxylin and eosin (H&E) staining and graded according to the following scale: 0, normal, no hepatocytes affected; 1, minor, <5% of hepatocytes affected; 2, mild, 5%-33% of hepatocytes affected; 3, moderate, 34%-66% of hepatocytes affected; and 4, severe, >66% of hepatocytes affected. Histological assessments of inflammation in the ileum were performed in a double-blind manner using H&E-stained sections. Histological scoring of the ileum was performed based on the severity of inflammation, the extent of injury, regeneration, and crypt damage. The final inflammation score for the ileum was calculated as the sum of the scores for all parameters. Inflammation was evaluated as follows: 0, normal; 1, mild increase in immune cell numbers and no colonic epithelial alterations; 2, a moderate increase in immune cell numbers and mild epithelial proliferation; and 3, severe increase in immune cell numbers and aberrant epithelial proliferation with extensive loss of villi/crypt architecture. Villus and crypt lengths in the ileum were determined according to an analysis of 15 well-oriented villi/crypts per mouse using the image analysis software LAS 2.0 (Leica). The inflammation scores or villus and crypt lengths were calculated as the medians of individual measurements from the two colonic sections of each mouse.

### Measurements of endotoxin levels

Serum endotoxin levels were analyzed using a commercially available endpoint LAL Chromogenic Endotoxin Quantitation Kit with a concentration range of 0.015-1.2 endotoxin units per milliliter (EU/mL; Thermo Fisher Scientific, Waltham, MA, USA), as previously described 29.

### Albumin enzyme-linked immunosorbent assay (ELISA)

Mice were transferred to individual cages 1 h prior to sacrifice to collect mouse feces for blood albumin leakage analysis. At the beginning of the sacrifice, one fecal pellet per mouse was immediately collected into an autoclave tube using sterile forceps. If there was no stool in the cage, the last feces from the colon closest to the rectum was collected and used for further analysis. One fecal pellet was used for albumin analysis. Feces were dissolved in sample dilution buffer (50 mM Tris, 0.14M NaCl, 0.05% tween20, pH 8.0) to a concentration of 100 mg/mL. Fecal albumin levels were quantified using a mouse albumin ELISA kit (Abcam, Cambridge, UK) according to the manufacturer's instructions. The results were subsequently quantified using a microplate reader (BioTek Instruments, Winooski, VT, USA). Human HGF was also analyzed using the HGF ELISA kit, and subsequently quantified using a microplate reader.

### Quantitative reverse-transcription polymerase chain reaction (qRT-PCR)

Total RNA was isolated from individual mouse livers and the small intestine (ileum), with overnight precipitation at -20 °C to increase the yield of RNA. Transcript levels were measured using the QuantStudio™ Real-Time PCR System (Applied Biosystems, Waltham, MA, USA) and commercial TaqMan probes (Applied Biosystems). The following cycling condition was used for qRT-PCR: 95 °C for 10 min, followed by 40 cycles of 95 °C for 15 s, 55 °C for 30 s, and 72 °C for 1 min. Data were normalized to GAPDH expression level, and the relative amount was calculated using the 2^-ΔΔCt^ method.

### Liver enterobacteria assay

To determine bacterial colonization in ethanol-exposed mice, liver tissues were collected at the indicated time points after binge ethanol exposure. For the colony-forming unit (CFU) assays, liver contents were weighed, homogenized in sterile PBS, serially diluted, and seeded on sheep blood agar plates cultured overnight at 37 °C under anaerobic conditions (Pack-Anaero; Mitsubishi Gas Chemical, Tokyo, Japan).

### Nitric oxide assay

Nitric oxide was measured as the amount of nitrite, which is the stable product of nitric oxide metabolism. Mouse sera were centrifuged at 4,000 rpm for 30 min, and the supernatants were collected and measured by a colorimetric assay using the Griess reaction (Invitrogen, Carlsbad, CA, USA). Sera of individual mice (100 μL) were incubated with an equal volume of Griess reagent at room temperature for 10 min. After incubation, the absorbance of the wells was measured at 550 nm using a microplate reader (Tecan, Männedorf, Switzerland).

### Evaluation of Barrier Function in Caco-2/tc7 Cells

Human colonic Caco-2/tc7 cells were maintained in Dulbecco's modified Eagle's medium (DMEM; Gibco, Rockville, MD, USA) containing 10% fetal bovine serum (FBS; Gibco) and penicillin/streptomycin. Caco-2/tc7 cells were seeded on polycarbonate membrane Transwell inserts with a surface area of 0.33 cm^2^ (0.4 μm pore size; SPL Life Science, Pocheon, Republic of Korea). After 1 d of cell seeding, the culture medium was replaced with fresh DMEM supplemented with 10% FBS and penicillin/streptomycin. The medium was changed three times throughout the week. After the trans-epithelial electrical resistance (TEER) exceeded 350 Ω cm^2^, intestinal barriers comprising Caco-2/tc7 cells were exposed to ethanol alone or co-treated with HGF for 24 h. TEER was measured using a Millicell Electrical Resistance System (ERS) meter (Millipore Corporation, Bedford, MA, USA). Data were collected from quadruple inserts per treatment in three experiments and expressed as a percentage of basal TEER obtained before ethanol treatment. At the end of the TEER measurements, 50 μg/μL FITC labeled 4-kDa dextran (FITC-D4; Sigma-Aldrich, St. Louis, MO, USA) was added to the apical side of the upper chamber of transwell plates seeded with Caco-2/tc7 cells. After 1 h of FITC-D4 treatment, FITC-D4 permeability to intestinal barriers was analyzed by spectrophotometrically measuring the fluorescent signals in the culture media of bottom wells using a 96-well microplate reader (Molecular Devices, San Jose, CA, USA) at excitation and emission spectra of 485 nm and 540 nm, respectively.

### Statistics

All statistical analyses were performed using the Mann-Whitney test (GraphPad Prism). Statistical significance was set at P < 0.05.

## Results

### Effect of SkMSCs on the body and spleen weights of binge ethanol-treated mice

**Figure 1:**
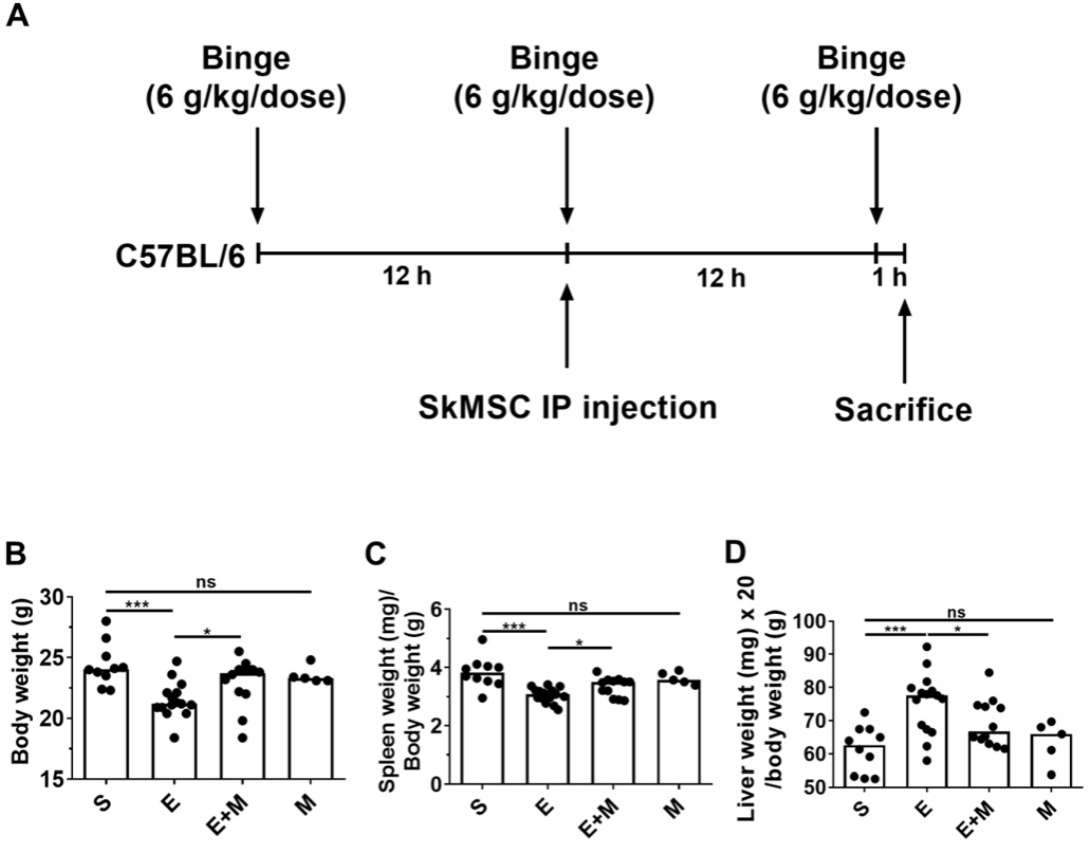
Experimental scheme and effects of skeletal muscle satellite cell-derived mesenchymal stem cells (SkMSCs) on body weight, spleen weight, and liver weight in binge ethanol-exposed mice. Eight-week-old C57BL/6 female mice were treated with binge ethanol and sacrificed on day 1. **(A)** Experimental design of the binge ethanol-induced liver injury model. **(B-D)** Body, spleen, and liver weight were measured after the third injection of ethanol. n = 5-17 mice per group. Scatter plot: horizontal bar, median. S, sham; E, binge ethanol; M, SkMSCs. Significance between treated groups was determined using Mann-Whitney U test. * *P* < 0.05, *** *P* < 0.001; ns, no statistical significance.

Binge alcohol exposure causes small intestinal injury, further mediating hepatic damage 28, which is associated with body weight loss 30. Further, alcohol consumption is known to be positively associated with functional hyposplenism 31-33. We examined the anti-inflammatory potential of SkMSCs in binge alcohol-exposed mice. C57BL/6 mice were orally administered three doses of 6 g/kg dose at 12-h intervals. Binge alcohol-exposed C57BL/6 mice were intraperitoneally injected once with SkMSCs (2.0 × 10^6^ cells/200 μL of HBSS) (Figure 1A). Mice were euthanized at the end of the third binge alcohol cycle, and their body and spleen weights were measured. Treatment with SkMSCs decreased the loss of body and spleen weights compared to treatment with binge alcohol alone (Figure 1B, C). Mice administered SkMSCs alone showed comparable body and spleen weights to those in the sham groups (Figure 1D), suggesting that SkMSCs alone do not overtly affect body and spleen weight.

### Effect of SkMSCs on liver damage in binge ethanol-treated mice

**Figure 2:**
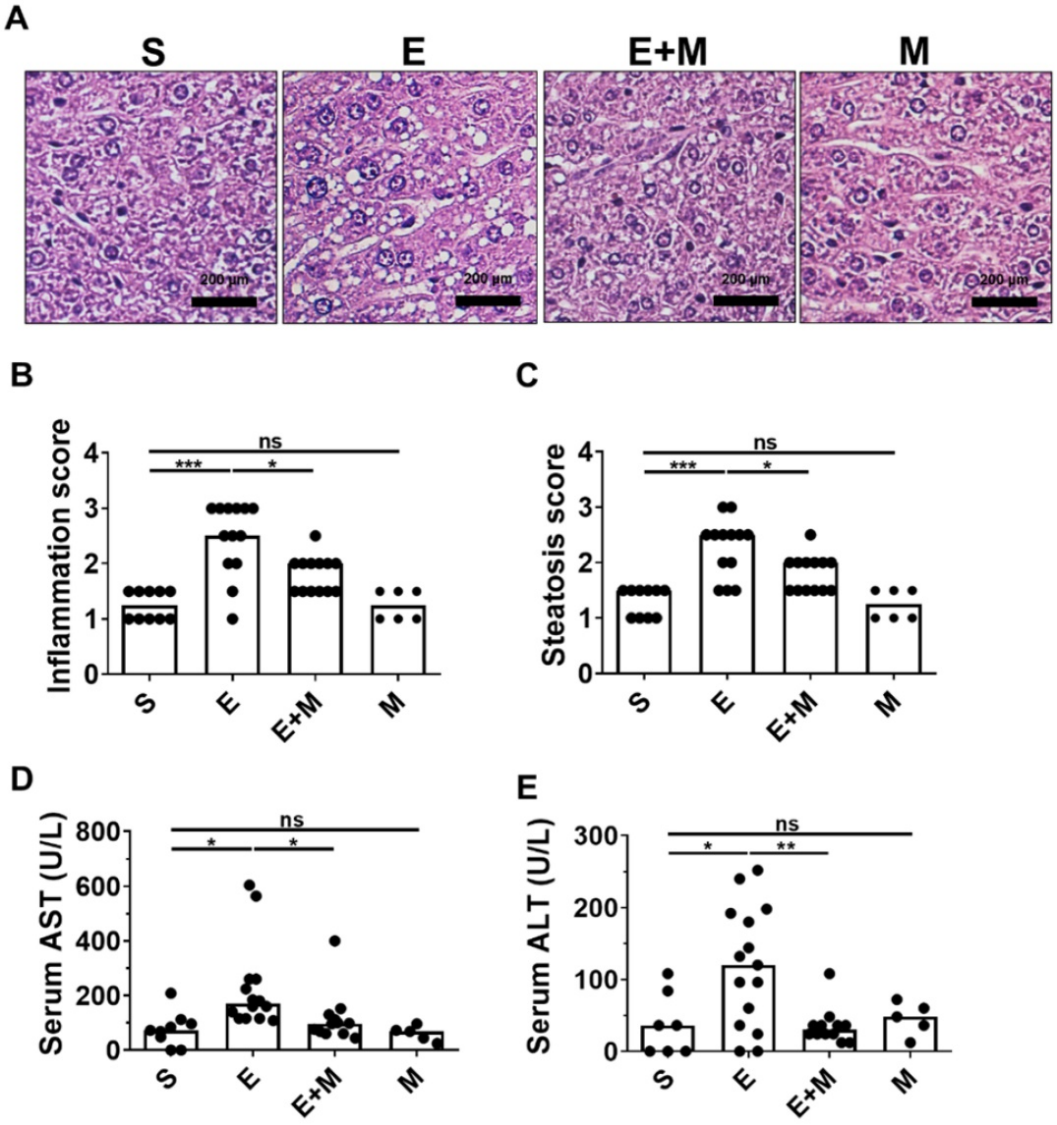
Effect of skeletal muscle satellite cell-derived mesenchymal stem cells (SkMSCs) on liver histology and serum AST and ALT levels in binge ethanol-treated mice. Eight-week-old C57BL/6 female mice were treated with binge ethanol. Mice were sacrificed on day 1, and liver tissues were fixed and stained with H&E. Representative images are shown. Histologic scores were evaluated using H&E slides. Serum AST and ALT levels were analyzed by a colorimetric assay kit. **(A)** H&E staining of the liver. **(B)** Inflammation score; **(C)** steatosis score; **(D)** serum AST levels; **(E)** serum ALT levels (n = 5-17 mice per group). Scatter plot: horizontal bar, median. S, sham; E, ethanol binge; M, SkMSCs. Significance between treated groups was determined using Mann-Whitney U test. * *P* < 0.05, *** *P* < 0.001; ns, no statistical significance.

Oral administration of binge ethanol induces histological hepatic inflammation and steatosis 27, 28. To determine whether treatment with SkMSCs could decrease ethanol-induced hepatic damage, mice were intraperitoneally administered SkMSCs, and hepatic inflammation and steatosis were histologically assessed at the end of the experimental period. Mice administered binge ethanol alone showed increased levels of inflammation and steatosis, as determined by H&E staining, whereas mice treated with binge ethanol + SkMSCs showed decreased levels of inflammation and steatosis (Figure 2B, C). Histologically, the administration of SkMSCs alone did not induce hepatic inflammation and steatosis in mice (Figure 2A-C). To determine whether SkMSCs could decrease the induction of liver damage, hepatic tissues from ethanol-treated mice administered SkMSCs were analyzed for serum AST and ALT levels. Based on the results, binge ethanol-treated mice administered SkMSCs had a significant decrease in serum AST and ALT levels compared to ethanol-treated mice (Figure 2D, E). Mice administered SkMSCs alone had comparable serum AST and ALT levels to those in the sham group, which suggests that SkMSCs alone do not induce liver damage. Collectively, these data indicate that SkMSCs ameliorate ethanol-induced hepatic damage.

### Effect of SkMSCs on bacterial translocation and inflammatory mediators in the small intestine of binge ethanol-treated mice

**Figure 3:**
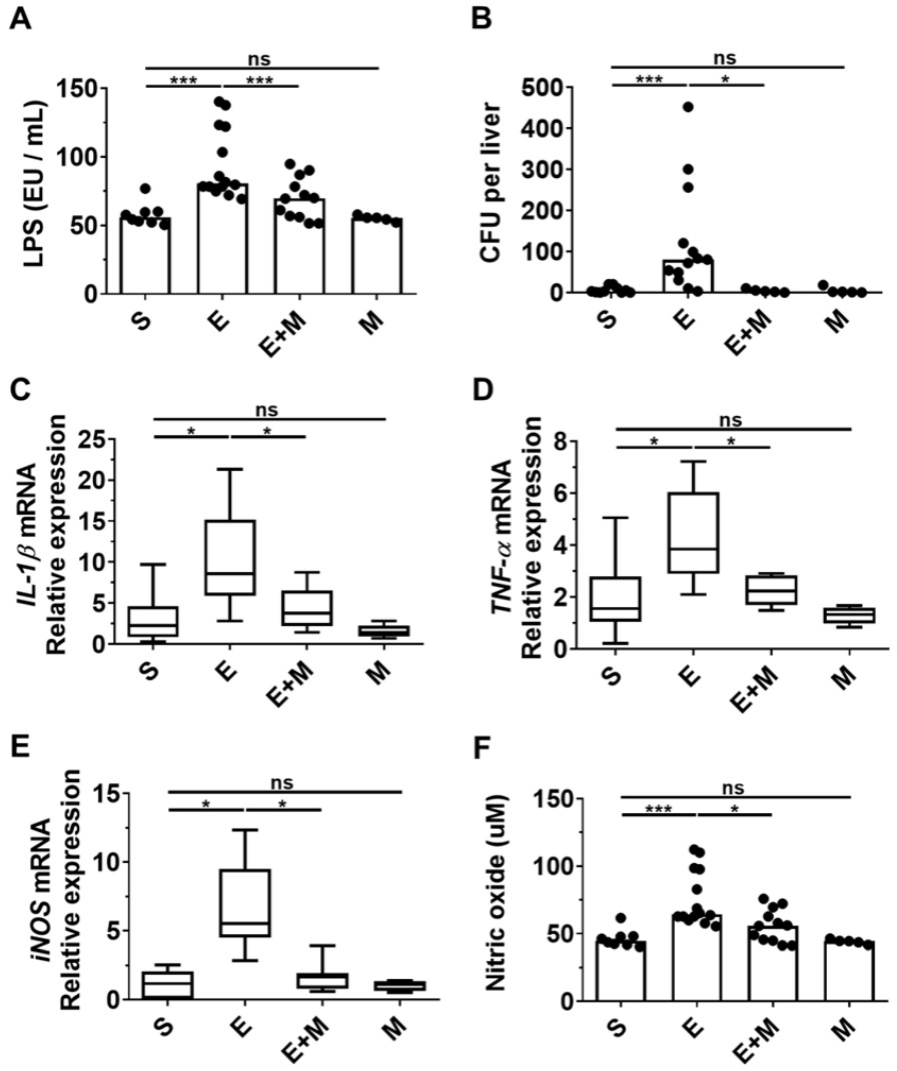
Effect of skeletal muscle satellite cell-derived mesenchymal stem cells (SkMSCs) on serum lipopolysaccharide (LPS), hepatic enterobacteria content, and pro-inflammatory genes in the liver tissues of binge ethanol-treated mice. Eight-week-old C57BL/6 female mice were treated with binge ethanol. Mice were sacrificed on day 1; serum and liver tissues were analyzed to determine LPS concentration using endotoxin activity assays and the mRNA expression levels of *IL-1β*, *TNF-α* and *iNOS* in the ileum were determined using qRT-PCR. Hepatic enterobacteria content in the liver tissues was analyzed using sheep blood agar plates. Sera of mice were analyzed to derive the nitrite content using nitric oxide assay kits. **(A)** Serum LPS levels; **(B)** average number of colonies in liver tissues; **(C)** *IL-1β* expression levels in the ileum; **(D)** *TNF-α* expression levels in the ileum; **(E)** *iNOS* expression levels in the ileum; **(F)** serum nitric oxide levels (n = 5-15 mice per group). Scatter plot: horizontal bar, median. S, sham; E, ethanol binge; M, SkMSCs. Significance between treated groups was determined using Mann-Whitney U test. * *P* < 0.05, *** *P* < 0.001; ns, no statistical significance.

Disruption of the intestinal barrier caused by binge ethanol promotes the translocation of bacterial products, such as LPS, to the liver, where they interact with hepatic Toll-like receptors (TLRs) and stimulate liver inflammation and damage 27, 28, 34. As SkMSCs decreased the generation of multiple lipid droplets in hepatocytes induced by binge ethanol (Figure 2A), as well as serum ALT and AST levels (Figure 2D, E), we hypothesized that they would decrease the translocation of LPS and intestinal bacteria, thereby suppressing inflammatory mediators, such as cytokines and reactive nitrogen species. To determine whether SkMSCs could decrease the translocation of bacterial products or gut microbes caused by ethanol binge drinking, serum and hepatic tissues of mice were analyzed for LPS and bacterial colonization using an endotoxin assay kit and anaerobic cultivation, respectively. The levels of LPS were found to be downregulated in ethanol-exposed mice treated with SkMSCs compared to mice treated with binge ethanol alone (Figure 3A, B).

Consistently, the CFUs were reduced in ethanol-exposed mice treated with SkMSCs compared to mice treated with binge ethanol alone (Figure 3A, B). Treatment with SkMSCs alone did not affect LPS levels in serum and CFUs in liver tissues, suggesting that they do not affect gut integrity. Additionally, small intestinal tissues (ileum) from binge ethanol-exposed mice administered SkMSCs were analyzed for *IL-1β*, *TNF-α*, and *iNOS* expression by qRT-PCR. Serum nitrite levels were also examined using a nitric oxide assay. Binge ethanol-exposed mice administered SkMSCs were found to display a decrease in *IL-1β*, *TNF-α*, and *iNOS* expression levels compared to those in mice treated with ethanol alone (Figure 3C-E). Nitrite (a metabolite of nitric oxide) levels in the serum were also decreased in ethanol-exposed mice administered SkMSCs, consistent with the iNOS expression data (Figure 3E), suggesting that SkMSCs decrease the binge ethanol-induced translocation of bacterial products and gut bacteria, simultaneously reducing the expression levels of inflammatory mediators. Collectively, these data support the hypothesis that SkMSC treatment decreases ethanol-induced hepatic damage and inflammation by suppressing the translocation of bacterial products and gut bacteria.

### Effect of SkMSCs on intestinal histopathology in binge ethanol-treated mice

**Figure 4:**
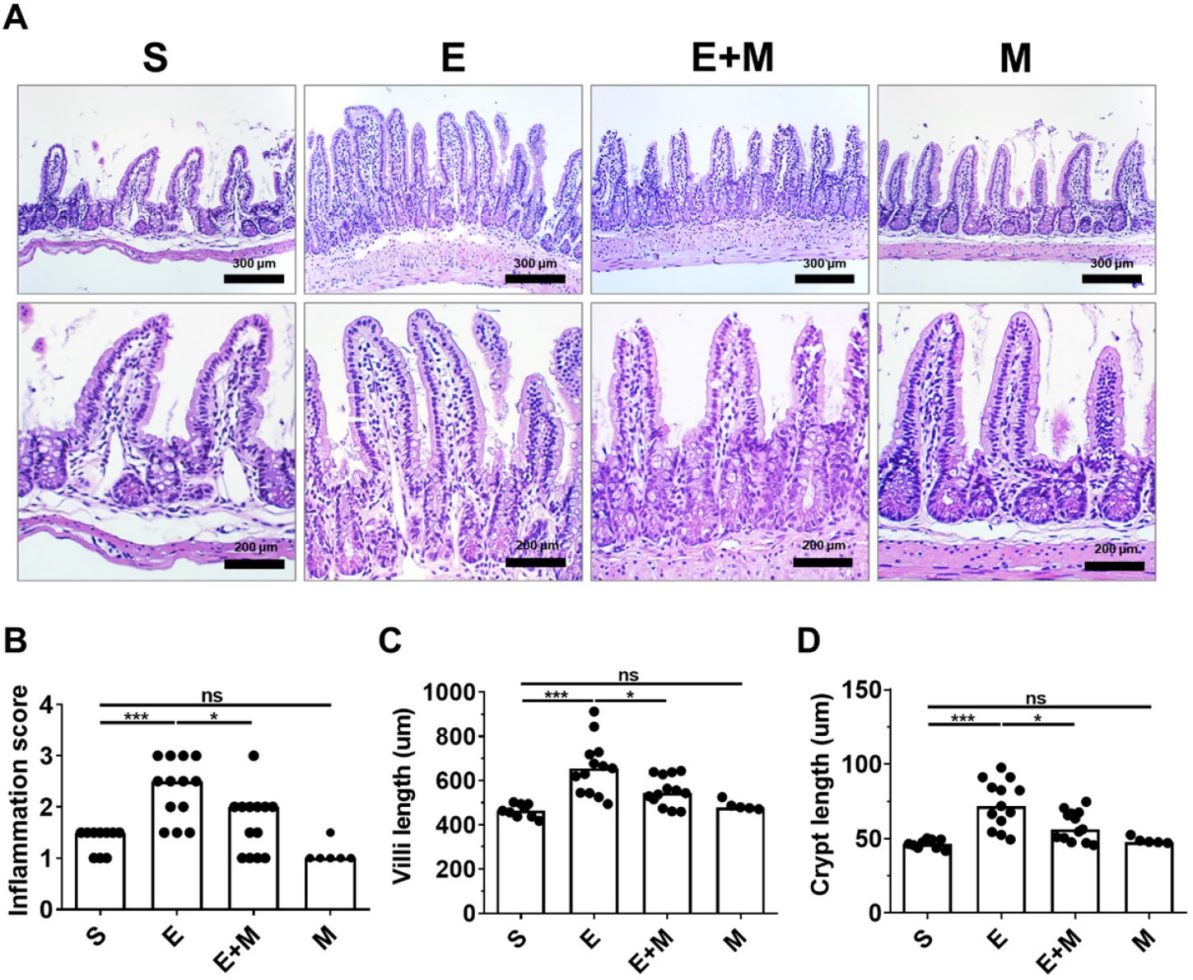
Effect of skeletal muscle satellite cell-derived mesenchymal stem cells (SkMSCs) on intestinal histopathology. Eight-week-old C57BL/6 female mice were treated with binge ethanol. Mice were sacrificed on day 1, and intestinal tissues (ileum) were fixed and stained with H&E. Representative images are shown. Histologic scores were evaluated using H&E slides. **(A)** H&E staining of small intestine (ileum); **(B)** inflammation scores; **(C)** villus lengths; **(D)** crypt lengths (n = 5-13 mice per group). Scatter plot: horizontal bar, median. S, sham; E, ethanol binge; M, SkMSCs. Significance between treated groups was determined using Mann-Whitney U test. * *P* < 0.05, *** *P* < 0.001; ns, no statistical significance.

Binge ethanol-induced hepatic injury is mediated by intestinal inflammation 27, 28. Moreover, binge ethanol-induced damage to the small intestine is characterized by changes in the length of the villi and crypts 35, 36. As SkMSCs decreased the translocation of LPS, bacteria, and inflammatory markers in the liver, it was further hypothesized that these cells could decrease ethanol-induced damage to the intestinal tissue (ileum). To test this hypothesis, tissue damage was assessed in the ileum via H&E staining. Ethanol-exposed mice administered SkMSCs showed reduced infiltration of inflammatory cells with diminished lengths of villi and crypt compared to mice administered binge ethanol alone (Figure 4A-D). No significant changes in inflammation scores and lengths of villi and crypts were observed between the sham and SkMSC-treated groups (Figure 4A-D), suggesting that treatment with SkMSCs does not affect intestinal histology. These findings suggest that SkMSCs decrease ethanol-induced hepatic injury by suppressing intestinal inflammation.

### Effects of SkMSCs on blood albumin leakage and inflammatory mediators in the liver of binge ethanol-treated mice

**Figure 5:**
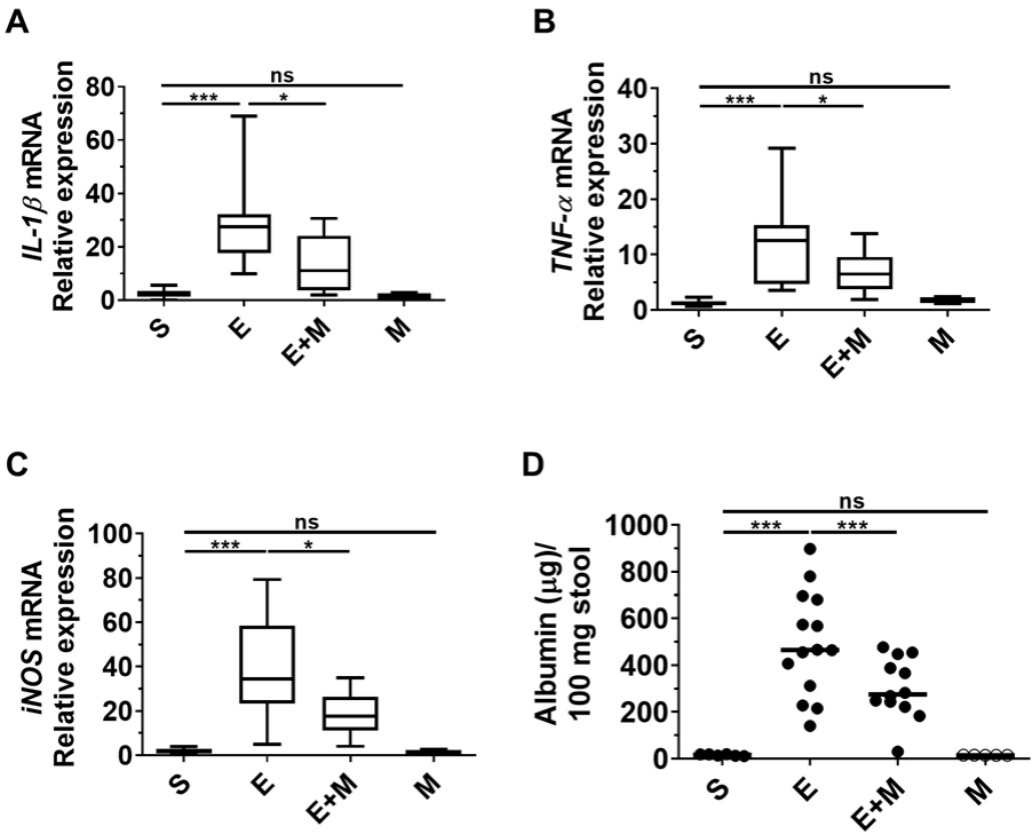
Effect of skeletal muscle satellite cell-derived mesenchymal stem cells (SkMSCs) on pro-inflammatory genes in the small intestine (ileum) and albumin levels in stool in binge ethanol-treated mice. Eight-week-old C57BL/6 female mice were treated with binge ethanol. Mice were sacrificed on day 1, and liver tissues were analyzed to determine *IL-1β*, *TNF-α*, and *iNOS* expression levels using qRT-PCR. The stool of mice was analyzed to detect albumin leakage by ELISA. **(A)** *IL-1β* expression; **(B)** *TNF-α* expression; **(C)** *iNOS* expression; **(D)** albumin levels in stool. (n = 5-12 mice per group). Scatter plot: horizontal bar, median. S, sham; E, ethanol binge; M, SkMSCs. Significance between treated groups was determined using Mann-Whitney U test. * *P* < 0.05, *** *P* < 0.001; ns, no statistical significance.

Inflammation of the small intestine induced by ethanol binge drinking disrupts gut permeability, which induces the leakage of albumin into the intestinal lumen from blood 28, 37, 38. As SkMSCs decreased binge ethanol-induced tissue damage in the small intestine based on histological assessments (Figure 4A-D), we proceeded to determine whether treatment with SkMSCs could diminish the expression of inflammatory cytokines in the liver using qRT-PCR. Ethanol-exposed mice administered SkMSCs showed reduced expression levels of *IL-1β*, *TNF-α*, and *iNOS* compared to those in mice administered ethanol alone (Figure 5A-C). Additionally, to test the barrier integrity of the small intestine, albumin was further examined in stool samples via ELISA. Binge ethanol-treated mice administered SkMSCs showed decreased levels of albumin compared to mice treated with ethanol alone (Figure 5D). Such finding suggests that SkMSCs decrease binge ethanol-induced liver inflammation by inhibiting intestinal inflammation.

### Effects of HGF on the intestinal epithelial cells, Caco-2/tc7

**Figure 6:**
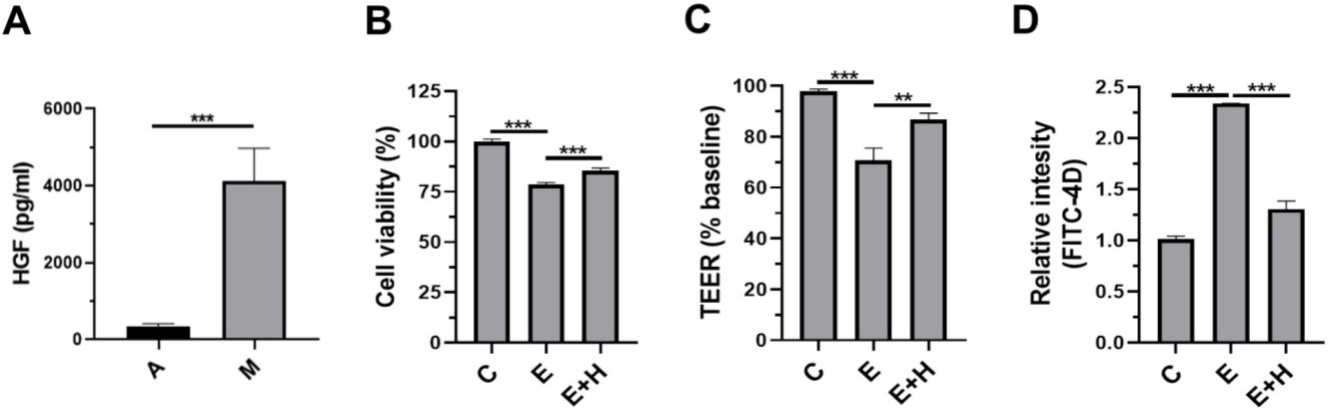
Treatment with HGF restores cell viability and permeability in ethanol-exposed intestinal epithelial cells. Human ASCs and SkMSCs were monocultured for 48 h. Thereafter, HGF applied to the culture supernatants of ASCs or SkMSCs was assessed via ELISA. Human intestinal Caco-2/tc7 cells were treated with HGF for 24 h. Cell viability was measured via the MTT assay. The barrier function of Caco-2/tc7 cells was measured for transepithelial electrical resistance (TEER) and translocated FITC-4k dextran **(A)** Secreted HGF in culture supernatants of ASCs or SkMSCs. **(B)** Cell viability of ethanol-exposed Caco-2/tc7 cells treated with HGF (10 ng/ml). **(C)** TEER of Caco-2/tc7 cells treated with HGF for 24 h. **(D)** Relative intensities of FITC-4k dextran in transwell seeded with Caco-2/tc7 monolayers treated with HGF for 24 h. A, ASCs; M, SkMSCs; C, control; E, ethanol; H, hepatocyte growth factor. Significance between treated groups was determined using Mann-Whitney U test. **P < 0.01, ***P < 0.001; ns, no statistical significance.

We sought to determine how SkMSC administration reduced alcohol-induced liver injury. Previously, we demonstrated that SkMSCs secrete more hepatocyte growth factor (HGF) than adipose tissue-derived mesenchymal stem cells (ASCs) 26. Before investigating the HGF-induced protective mechanisms, we checked the basal level of HGF in the culture supernatants of SkMSCs. As expected, SkMSCs significantly produced HGF (Figure 6A). As binge alcohol-induced liver injury is mediated by disruption of the epithelial barrier, we hypothesized that HGF directly protects against ethanol-induced damage to the intestinal barrier. To test this hypothesis, the human intestinal epithelial cell line, Caco-2/tc7 cells, was co-treated with 40 mM ethanol and HGF (20 ng/mL) for 24 h. Thereafter, viability assay (MTT assay), permeability test (trans-epithelial electrical resistance [TEER] analysis, and FITC-4k dextran assay [FITC-D4] were conducted. Ethanol-exposed Caco-2/tc7 cells displayed decreased cell viability compared to control cells (Figure 6B). Further, Caco-2/tc7 cells co-treated with 40 mM ethanol and HGF (20 ng/mL) for 24 h showed increased cell viability compared with Caco-2/tc7 cells treated with 40 mM ethanol alone (Figure 6C). Consistently, treatment with HGF recovered the decrease in TEER and increased the permeation of FITC-D4 (Figure 6C, D). These results indicate that the protective roles of SkMSCs in ethanol-induced liver injury and gut leakiness are mediated by HGF-induced protection of intestinal epithelial cells against ethanol-induced damage.

## Discussion

To our knowledge, the present study is the first to demonstrate the anti-inflammatory effects of SkMSCs on ethanol-induced hepatic and intestinal inflammation. Histological analysis revealed decreased extent of inflammation in the small intestines of SkMSC-treated ethanol-fed mice compared to that in binge ethanol-treated mice. Moreover, inflammation-associated cytokine levels were reduced in the livers and intestines of SkMSC-treated ethanol-fed mice. We hypothesize that the SkMSC-induced suppression of hepatic injury in the binge ethanol model was mediated by diminishing intestinal inflammation.

Intestinal inflammation induced by ethanol or fructose involves the destruction of the intestinal barrier, which in turn leads to gut leakiness 27, 28, 34. Gut leakage induced by ethanol binding *in vivo* may be directly caused by ethanol itself. For example, ethanol (80 mM) treatment induces the downregulation of barrier function by increasing the levels of CYP2E1/reactive oxygen species- or iNOS/reactive nitrogen species-mediated protein degradation of tight junctions in T84 or Caco-2 intestinal epithelial cells 28, 39-41. As FAPs can migrate to inflamed tissues enriched with CXCL via the CXCR receptor 42, 43, their migration might be specifically effective in the targeting of intestinal epithelial cells in the inflamed gut by downregulating the expression of CYP2E1 or iNOS in a paracrine manner.

The compromised integrity of the gut barrier is characterized by increased levels of fluorescein isothiocyanate-dextran, LPS, and fecal albumin, which are indications of increased intestinal permeability based on several animal studies 44-46. Diet-related animal studies have revealed the critical role of the intestinal barrier in preventing liver inflammation due to the translocation of bacterial products or gut bacteria 34, 47, 48. However, we did not investigate permeability with fluorescein isothiocyanate-dextran in a binge ethanol model after the administration of SkMSCs *in vivo*. However, we revealed elevated levels of LPS and albumin in the serum and stool (Figure 3A, B, and Figure 5D). Further, HGF treatment was found to rescue ethanol-induced barrier disruption *in vitro* (Figure 6). HGF might inhibit CYP2E1-mediated nitro-oxidative stress by ethanol in intestinal epithelial cells, thereby suppressing the degradation of junctional proteins. Further studies are required to understand the mechanism of SkMSC/HGF in detail.

Hepatic inflammation and steatosis (an accumulation of lipid droplets) are promoted by the translocation of LPS from gram-negative bacteria in several models of fatty liver diseases 49-51. LPS is a ligand for TLR4 receptors on innate immune cells, including neutrophils and macrophages 52, 53, and induces IL-1β, TNF-α, reactive oxygen species, and reactive nitrogen species, which trigger hepatic injury and steatosis 54, 55. In our study, we did not investigate infiltrated neutrophils or macrophages in the hepatic tissues of mice treated with binge ethanol or SkMSCs. However, treatment with SkMSCs may directly decrease the activation of LPS-activated neutrophils or macrophages. Additional studies are required to discern the SkMSC-mediated mechanism underlying the suppression of ethanol-induced liver injury in terms of lipogenesis.

Previously, we performed an analysis of the transcriptome of SkMSCs using next generation sequencing and revealed that the expression levels of COX-2 and HGF were significantly increased in SkMSCs co-cultured with IFN-γ- and LPS-treated THP-1 macrophages 26. COX-2 leads to an increase in PGE2 level 56, 57 for the mediation of an anti-inflammatory neutrophil phenotype at sites of tissue injury *in vivo* 58. PGE2 has also been reported to induce M2 polarization of macrophages in inflammatory tissues 59, 60. Secreted PGE2 or HGF has also been found to suppress the inflammatory status of M1 macrophages *in vitro* and *in vivo* 61, 62. Specifically, Choi et al. showed that HGF level increases the transition from M1 macrophages to the M2 phenotype through activation of AMPK signaling and a decrease in the M1 phenotype-associated inflammatory cytokines (IL-1β, TNF-α, and iNOS) in an LPS-treated murine macrophage cell line 61. Herein, we did not investigate AMPK-related signaling in macrophages. PGE2 or HGF by SkMSCs might decrease ethanol-induced hepatic injury by suppressing inflammatory M1 macrophages or promoting anti-inflammatory M2 macrophages; this is because alcoholic liver diseases are exacerbated by inflammatory neutrophils and macrophages. In addition to the anti-inflammatory effects of PGE2 and HGF, PGE2 and HGF have been reported to promote the regeneration of intestinal epithelial cells 63, 64. In a previous study, SkMSCs co-cultured with macrophages produced both PGE2 and HGF; however, SkMSCs cultured alone expressed only HGF at high levels. Therefore, in this study, the effect of HGF on the intestinal epithelial cells, Caco-2/tc7, was analyzed *in vitro*. Based on our findings, HGF protects intestinal epithelial cells from alcohol-induced damage. As PGE2 can also act on the regeneration of intestinal epithelial cells, both PGE2 and HGF produced by SkMSCs may reduce intestinal inflammatory responses and have protective effects on intestinal epithelial cells in acute alcohol-induced liver injury. As the direct mechanism of HGF and PGE2 production by SkMSCs has not been validated in a model of acute alcohol-induced liver injury, our study had limitations.

Taken together, our study provides evidence that SkMSCs can be used to inhibit ethanol-induced liver injury, potentially by reducing levels of intestinal inflammation. Although the ability of SkMSCs to diminish tissue inflammation is expected, our study unveils the potential strategy of targeting intestinal inflammation to prevent ethanol-induced liver damage. Future studies would be critical to evaluate the therapeutic efficacy of SkMSCs in gut-mediated chronic inflammatory diseases *in vivo*.

## Conclusions

This study revealed the anti-inflammatory effects of human SkMSCs in an ethanol-induced liver injury model. Treating human skeletal tissue with SkMSCs suppressed hepatic inflammation and injury by reducing gut inflammation and leakiness caused by excessive ethanol intake. These findings may prove useful to assess the outcomes of future SkMSC-based therapies in humans with alcoholic liver diseases.
